# Correlation between the ERD in grasp/open tasks of BCIs and hand function of stroke patients: a cross-sectional study

**DOI:** 10.1186/s12938-023-01091-1

**Published:** 2023-04-15

**Authors:** Jianghong Fu, ZeWu Jiang, Xiaokang Shu, Shugeng Chen, Jie Jia

**Affiliations:** 1 Department of Rehabilitation Medicine, Huashan Hospital, Fudan University, 12 Mid-Wulumuqi Road, Jing’an District, Shanghai, 200040 China; 2 School of Mechanical Engineering, Shanghai Jiao Tong University, Shanghai, China; 3 National Clinical Research Center for Aging and Medicine, Huashan Hospital, Fudan University, Shanghai, China; 4 National Center for Neurological Disorders, Shanghai, China

**Keywords:** Grasp, Open, Brain–computer interfaces, Stroke, Motor task

## Abstract

**Background and aims:**

Brain-computer interfaces (BCIs) are emerging as a promising tool for upper limb recovery after stroke, and motor tasks are an essential part of BCIs for patient training and control of rehabilitative/assistive BCIs. However, the correlation between brain activation with different levels of motor impairment and motor tasks in BCIs is still not so clear. Thus, we aim to compare the brain activation of different levels of motor impairment in performing the hand grasping and opening tasks in BCIs.

**Methods:**

We instructed stroke patients to perform motor attempts (MA) to grasp and open the affected hand for 30 trials, respectively. During this period, they underwent EEG acquisition and BCIs accuracy recordings. They also received detailed history records and behavioral scale assessments (the Fugl-Meyer assessment of upper limb, FMA-UE).

**Results:**

The FMA-UE was negatively correlated with the event-related desynchronization (ERD) of the affected hemisphere during open MA (*R* = − 0.423, *P* = 0.009) but not with grasp MA (*R* = − 0.058, *P* = 0.733). Then we divided the stroke patients into group 1 (Brunnstrom recovery stages between I to II, *n* = 19) and group 2 (Brunnstrom recovery stages between III to VI, *n* = 23). No difference during the grasping task (*t* = 0.091, *P* = 0.928), but a significant difference during the open task (*t* = 2.156, *P* = 0.037) was found between the two groups on the affected hemisphere. No significant difference was found in the unaffected hemisphere.

**Conclusions:**

The study indicated that brain activation is positively correlated with the hand function of stroke in open-hand tasks. In the grasping task, the patients in the different groups have a similar brain response, while in the open task, mildly injured patients have more brain activation in open the hand than the poor hand function patients.

## Background

Stroke causes the highest morbidity associated with disability-adjusted life years lost in China, with 2 million new cases annually [1]. Up to 66% of stroke survivors experience upper limb motor impairments, which result in functional limitations in activities of daily living and decreased life quality [2, 3]. Hand function rehabilitation is a hotspot and challenge in the field of neurological rehabilitation. Brain-computer interfaces (BCIs) have been emerging for years, and they have been proven to be effective for hand recovery in different stages of stroke patients [4–6].

BCIs allow for device control without motor involvement as they translate brain activation into output signals for communication or environmental control [7], and the sensorimotor rhythm (SMR) based BCIs detect characteristic changes in SMR in response to the motor task. The SMR based on BCIs training was adopted in several studies [8–10]. SMRs can be measured over the sensorimotor cortex (SMC) and modulated by motor imagery (MI), motor attempt (MA), or motor execution (ME) tasks [11, 12]. Task-related modulation in EEG-based SMRs is usually manifested as event-related desynchronization (ERD) or event-related synchronization (ERS) in low-frequency components [mu rhythm (8–12 Hz) and beta rhythm (13–26 Hz)] [13]. MI, MA, or ME is associated with ERD of mu rhythm oscillations recordable over SMC (electrode sites C3 and C4 according to the 10/20 system) using EEG [14, 15]. ERD usually appears when SMR decreases, it happens in active motion, such as MI, while ERS means that SMR increases, which usually happens in the termination of movement or MI [16]. MI is a mental activity, in which a specific movement is performed in the mind without actual movement [17]. MA is an attempt of the paralyzed limb to move while there is still no actual or little movement but the electromyography activity in the affected arm was several orders higher in the motion phase than in the rest phase [18]. They were both used extensively in the BCI experiment as an active way of neuromodulation. ME was mostly used in healthy subjects [19, 20]. Specifically, MA-based BCI is a better choice for stroke rehabilitation [21], and patients can easily understand the instruction and perform the MA tasks.

However, the type of motor tasks in BCIs experiments were not so abundant nowadays, not only limited by the EEG recognition technology but also the various levels of motor impairments of stroke patients. Many of them in the acute stage undergo flaccid paralysis stage, and they can hardly move their hands, even to do an incomplete grasp. Lots of patients may go through the synergetic motion mode stage, where they can perform different levels of grasping activity. However, the segregation movement is a harder step, so the full extension of affected fingers means great progress in recovery, and the process conforms to the recovery of the I–VI Brunnstrom recovery stages [22]. Imaging the grasp [23] or/and extension [24] of the affected hand were designed in many research and some movements were combined with the activity of daily living. Ramos-Murguialday [25] trained patients to move the upper arm and reach forward with the help of arm orthosis. Patients were instructed to try to reach, grasp, and bring an imaginary apple to their lap, and finger extension is involved in reaching and grasping movement. Therefore, the motor tasks usually focused on the paretic limb. The common motor tasks adopted in the BCIs experiment included the movements of the upper joint, shoulder, elbow, wrist, and hand, especially the distal joint. The hand tasks consisted of many forms, both simple and complex, which include not only basic movements but also the movements of daily life. But few of them have applied the different motor tasks to BCIs experiments and explored the combined motor task to promote motor recovery after a stroke.

Previous studies indicated that brain activation may correlate with motor recovery from stroke [16, 24, 26]. A higher magnitude of ERD activity is related to larger cortical activation during motor tasks. ERD/ERS is triggered by the voluntary movement of the hemiparetic hand [27], movement-related beta ERD over the affected SMC has been investigated in many studies [28, 29]. ERD in the alpha band (α-ERD, frequencies ranging from 8 to 13 Hz) has been hypothesized to reflect cortical activation or disinhibition. The α-ERD has been observed not only during the elaboration of stimuli belonging to different sensory modalities (i.e., sensory-related α-ERD) but also during various mental tasks (i.e., task-related a-ERD) [30]. We also found that there was a positive correlation between the Fugl-Meyer assessment of upper limb (FMA-UE) and ERS in the unaffected hemisphere in MI tasks, and there were negative correlations between FMA-UE scores and ERD in both hemispheres [31] in MA task. However, the relationship between the different motor tasks performed by the various hand function of a stroke patient and the brain activation is still unclear.

In practice, the Brunnstrom stage can describe the hand function from a qualitative aspect, while the FMA-UE was used more as a scale to quantify motor impairment, and it is of better reliability and validity [32]. Therefore, we hypothesized that brain activation is correlated with the FMA-UE and different motor tasks. In specific, a better functional status may have more brain activations when performing hand extension than grasp. Thus, our research is focused on FMA-UE and explores brain activations when they perform the two different motor tasks.

## Results

Forty-seven stroke patients were enrolled in the experiment, of which 5 were excluded because they didn’t finish the whole evaluation or the EEG data was ruined, and 42 were used for analysis. The characteristic of the patients from the two groups is shown in Table 1.

**Table 1:** The characteristic of the patients from the two groups

Item	Group 1 (*n* = 19)	Group 2 (*n* = 23)	*t*/χ^2^/*Z*	*P* values
Age (MD ± SD)	59.21 ± 11.23	54.04 ± 12.06	1.425	0.162
Male/(%)	14 (73.7%)	17 (73.9%)	0.000	> 0.999
Type			0.505	0.477
Infarction	13 (68.4%)	19 (82.6%)		
Hemorrhage	6 (31.6%)	4 (17.4%)		
Course/days	80.00 (42.00, 121.00)	68.00 (26.00, 174.00)	− 0.531	0.596
MMSE	27.00 (24.00, 29.00)	28.00 (26.00, 30.00)	− 1.357	0.175
FMA-UE	10.16 ± 3.82	33.35 ± 13.40	− 7.917	< 0.001

The data conformed to Gaussian distribution were been demonstrated by MD ± SD, or they were demonstrated by M (P25-P75). *MMSE* mini-mental state examination, *FMA-UE* the upper limb of the Fugl-Meyer assessment

**Fig. 1:**
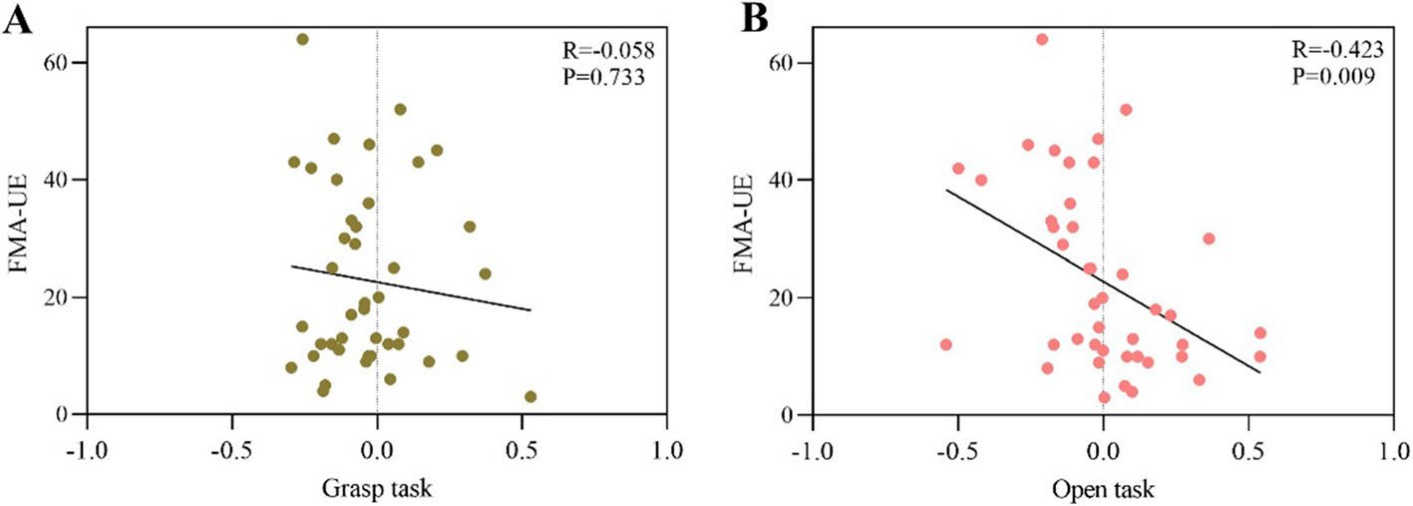
**A** The correlation between ERD in the affected hemisphere in grasp tasks and FMA-UE for all patients. **B** The correlation between ERD in open tasks and FMA-UE for all patients

For all the patients, after gender, age, diagnosis, MMSE, and course of disease were set as covariables, the partial correlation between the ERD of the affected hemisphere during open MA with the FMA-UE was negatively (*R* = − 0.423, *P* = 0.009) while the ERD of the affected hemisphere during grasp MA with the FMA-UE was not statistically significant (*R* = − 0.058, *P* = 0.733) (Fig. 1). On the unaffected hemisphere, there was no significant correlation between the FMA-UE and ERD during grasp MA (*R* = − 0.083, *P* = 0.626) or open MA (*R* = − 0.257, *P* = 0.125).

**Fig. 2:**
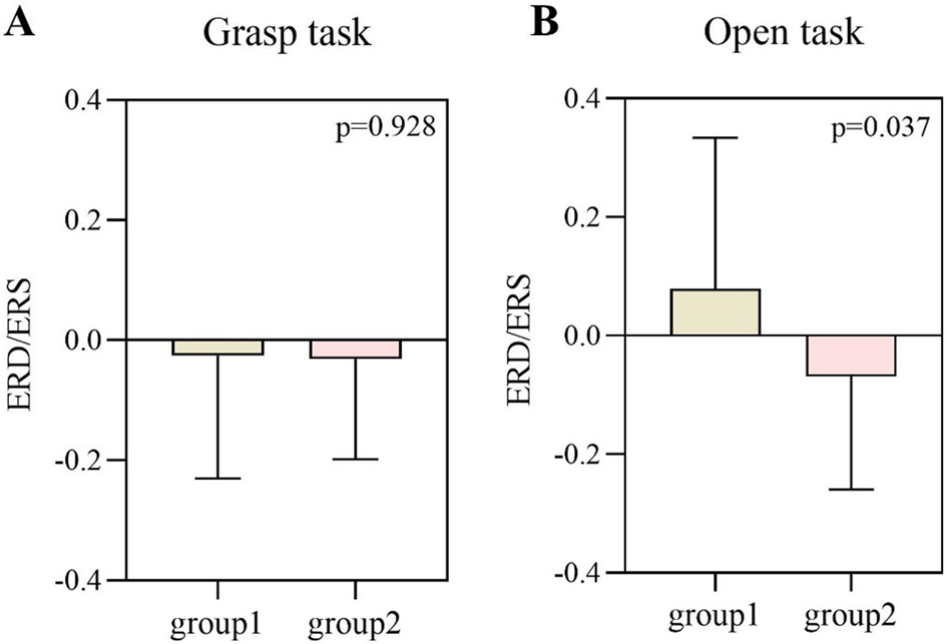
The comparison of ERD in the affected hemisphere for the different tasks and groups with a histogram. **A** The comparison of ERD between group 1 and group 2 in the grasping task. **B** The comparison of ERD between group 1 and group 2 in the open task

**Fig. 3:**
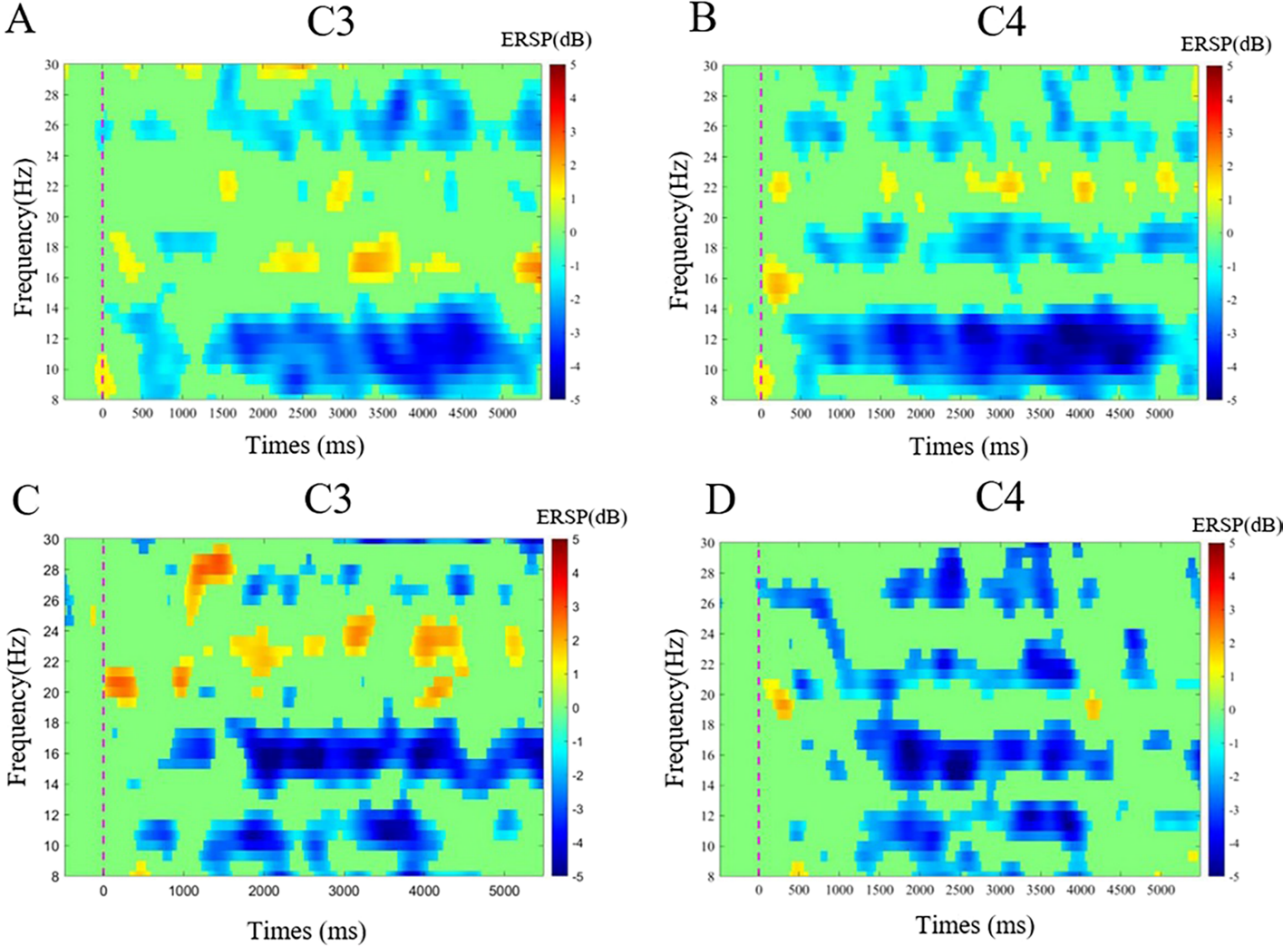
Example of the one-stroke patient’s brain activation in different experimental conditions from group 1 with poor hand function had a left brain injury. **A**, **B** Time–frequency graph of ERSP values in grasp tasks of C3/C4 channels; **C**, **D** Time–frequency graph of ERSP values in open tasks of C3/C4 channels. At 0 s, the voice instruction prompts the patient to prepare for the motor task, and at 3–5 s, the patient is performing the motor task

**Fig. 4:**
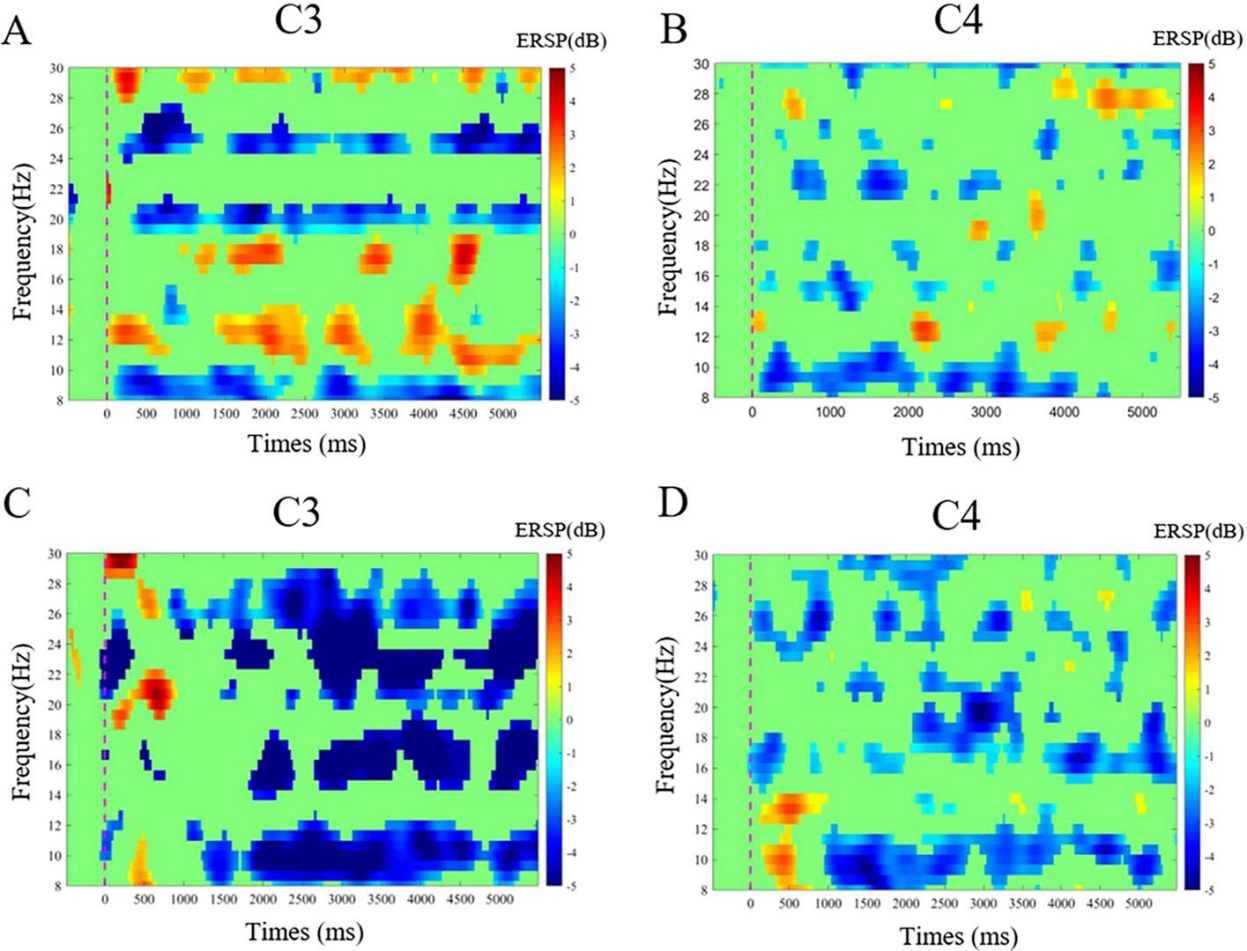
Example of the one-stroke patient’s brain activation in different experimental conditions from group 2 with a relatively better hand function which had a left brain injury. **A**, **B** Time–frequency graph of ERSP values in grasp tasks of C3/C4 channels; **C**, **D** Time–frequency graph of ERSP values in open tasks of C3/C4 channels. At 0 s, the voice instruction prompts the patient to prepare for the motor task, and at 3–5 s, the patient is performing the motor task

As for the two tasks on the affected hemisphere, in the open task, the ERD of group 1 was 0.0789 ± 0.2546, and the ERD of group 2 was − 0.0693 ± 0.1905, there was a significant difference between the two groups (*t* = 2.156, *P* = 0.037). As for the grasping task, the ERD of group 1 was − 0.0258 ± 0.2049, and the ERD of group 2 was − 0.0310 ± 0.1672, and there was no difference between the two groups (*t* = 0.091, *P* = 0.928) (Figs. 2, 3, 4). On the unaffected hemisphere, there is no significant difference between the two groups whether in the grasp MA (*t* = − 1.199, *P* = 0.237) or open MA (*t* = 1.297, *P* = 0.202).

In addition, patients over 65 years of age (10 patients) were excluded and the statistical analysis was re-performed. The correlation between ERD of the affected hemisphere during open MA with the FMA-UE was statistically significant (*R* = − 0.502, *P* = 0.003), while, they were not correlated with the FMA-UE on the affected hemisphere during grasp task (*R* = 0.024, *P* = 0.895). And on the unaffected hemisphere, there is still no significant correlation between the ERD under grasp MA (*R* = 0.008, *P* = 0.967) or open task (*R* = − 0.265, *P* = 0.143) with FMA-UE. For the subgroup analysis, the ERD was a significant difference (*t* = 2.073, *P* = 0.047) between group 1 (*n* = 12) and group 2 (*n* = 20) during the open task on the affected hemisphere, while the rest task shows no significant results.

For all patients, the BCI accuracy during both grasp (*R* = 0.231, *P* = 0.168) or open MA (*R* = − 0.058, *P* = 0.733) was not correlated with the ERD of the affected hemisphere, or with the FMA-UE. In addition, the BCI accuracy between the two groups was (*t* = − 0.982, *P* = 0.332) for the grasping task, and the BCI accuracy for the open task was (*t* = 0.607, *P* = 0.547), no significant difference was found for the two groups whether they perform the grasp or open task.

## Discussion

This study compared the ERD in stroke patients when they performed the different motor tasks in the BCI system, as the motor tasks assigned were attempts to grasp and open the hand. And we found the ERD in the affected hemisphere during the open task was negatively correlated with the FMA-UE. The clinical recovery of paretic hand function is believed to occur via the affected corticospinal plasticity and vicarious recruitment of widespread frontal and parietal synergistic regions [34], thus we mainly focus on the brain electrical activity of the affected hemisphere, and the C3/4 is used extensively as the hand representation in the brain [4]. In our study, we focus on the ERD of the affected hemisphere from C3/4 and explored brain activation with the FMA-UE. The FMA-UE of the patients enrolled in our study varied from 4 to 64, some patients may have severe hand function impairment, thus some ERS appeared on the affected hemisphere because the unaffected participated more than the affected hemisphere. Vera Kaiser et al. classified the relationship between ERD and ERS patterns and the degree of stroke impairment. They found higher impairment was related to stronger ERD in the unaffected hemisphere and higher spasticity was related to stronger ERD in the affected hemisphere, and both were related to a relatively stronger ERS in the affected hemisphere [33]. In our study, the ERD of the unaffected hemisphere during open MA was negatively correlated with the FMA-UE (*R* = − 0.307, *P* = 0.048) before these covariates were set. Thus, some covariates can affect the results, especially mental stages, and age. Expect that, we reached similar results compared to previous study [31].

The activity of the SMR is indicative of the brain’s responsiveness to an excitatory drive and reflects the current excitatory state [35, 36], and some studies have testified the ERD magnitude corresponds to corticospinal excitability increases in healthy subjects and patients with hemiplegic stroke [36]. Therefore, we chose the ERD to reflect the brain activation in the different motor tasks. We compared the FMA-UE with the ERD in 8–13 Hz and found the ERD of the open task is negatively correlated with FMA-UE, but not with the grasping task. Different EEG characteristics of different tasks have been explored by many researchers. Some scholars [37] have discriminated different reach-and-grasp actions through EEG parameters. As is knowing, grasp is the original movement of human beings. Whereafter, people learn how to open their fingers and drop an object as planned. Especially, hand opening is a necessary condition for complete hand functionality. In our research, even though there was no significant difference between the ERD during the grasping task with the FMA-UE or brain activation difference between group 1 and group 2, the ERD of group 2 was still lower than group 1, and it may need more research. Grasp is the most fundamental function of the hand. People may have a similar reaction to the basic movement, and this leads to the brain activation of severely injured patients being as strong as that of mildly to moderately injured patients. While in the open task, the ERD of group 2 was statistically significantly lower than that of group 1. It may reflect that the moderately to mildly injured brain may be more sensitive to open movement, and a relatively higher preserved brain function may be more suitable to be trained in the separation movement or complex movement.

One of the recovery mechanisms of BCIs related to motor tasks is motor learning. Not only Wadden. et al. [40] found a faster rate of motor sequence acquisition from healthy people to stroke, but some researchers [41] also pointed out that some brain regions are related to motor control or motor learning, including the cerebellum, parietal cortex, premotor cortex, motor cortex, and basal ganglia. Haar assumed that [42] posterior parietal cortex, somatosensory cortex, premotor cortex, and motor cortex represent task state, body state, task action, and body action, respectively. A brain is a general commander. The linkage of multiple brain regions is necessary to integrate multiple functions. Some severe injuries may damage the integrated brain systems, resulting in motor learning impaired. Some scholars [43] have demonstrated the affected hand has an execution ability impaired, and stroke patients have impaired anticipatory scaling of grip force and load force rate to the object weight in a grasping task. It turns out that the more brain region damaged, the more motor learning is damaged and the less hand function is preserved. Therefore, more motor tasks designed in BCI experiments, and brain reactions to different tasks, combined with their recovery process, are worth further research.

The recovery of the stroke patient obeys the rules talked about above. FMA-UE is used extensively and is of high reliability and validity when measuring the functional status of stroke patients [32]. We adopted the FMA-UE as a reflection of the brain injury and the functional status of the upper limb. Some research has shown that the recovery of motor function may be through activation of the motor cortex of the affected hemisphere [44–46], so with more activation of the brain, the task allocated to the patients may promote their recovery greatly. We choose the FMA-UE as the indicator for motor impairment, considering kinds of confounding factors that may influence the results, such as sex, age, diagnosis (infarction/hemorrhage), the disease of course, and MMSE, they were all set as covariates when calculating the correlation analysis.

Our studies show that BCI accuracy does not correlate with the activation of the affected hemisphere or the functional status of patients. BCI accuracy reflects the ability to interact between the human brain and the computer device. People can control the BCIs equipment mainly depending on its adaptation to the interaction. Though patients with better recovery showed relatively higher online BCI accuracy [47], some studies also didn’t prove a positive correlation between recovery with BCI accuracy [6]. However, some studies have shown that proprioceptive feedback (feeling and seeing hand movements) improved BCI accuracy [48], so researchers may pay more attention to the BCI accuracy with the motor impairment or the brain activation, and promote patients adapted to the interaction system may benefit their rehabilitation process.

### Limitations and future work

First of all, a bigger sample size is needed. Then, the EEG acquisition equipment was 10 channels. It has two-sided, so it is easier to record the electrical activity of a patient's brain, but the 10 channels limit the study of more brain regions and make further EEG analysis, such as functional connectivity of the whole brain.

Our study is a basic concept motor task exploration of a BCI experiment. Based on the results, we can assign the motor task properly, and give them the task which can activate their brain as much as possible, to obtain a greater degree of functional recovery. And we have already set about the clinical experiment to observe how much BCIs will help patients recover.

## Conclusion

The study indicated that brain activation is positively correlated with the hand function of stroke in open-hand tasks. In the grasping task, the patients in the different groups have a similar brain response, while in the open task, mildly injured patients have more brain activation in open the hand than the poor hand function patients. It may be indicated a related preserved motor function may be more suitable to be trained in the separation movement. Therefore, it may evoke us to notice what kind of BCI paradigms should be designed and what kind of motor task should be assigned to the patients.

## Materials and methods

### Research patients/subject recruitment

We conducted a cross-sectional study, and by one-time evaluation of stroke patients. Patients in the subacute or chronic stage of stroke were recruited from the Department of Rehabilitation Medicine, Huashan Hospital from Feb.2021 to Dec.2021.

The inclusion criteria for patients following stroke were (1) ischemic or hemorrhagic stroke diagnosed through computed tomography or MRI; (2) age in the range of 18–80 years; (3) at least 2 weeks since stroke onset and less than one year; (4) mini-mental state examination ≥ 20 scores, ability to obey the basic command; and (5) ability to sit on a chair independently for at least 1 h. The exclusion criteria were (1) having a cardiac pacemaker; (2) pregnancy; (3) allergy to EEG electrode cream; (4) any osteoarthrosis (including joint deformity) that could cause joint contracture in the hand or upper limb; and (5) unstable fracture in the paretic upper limb. Written informed consent was provided by all participants. This study was approved by the ethical committee of Huashan Hospital [(2021) Provisional Examination No. (039)] and was performed according to the Declaration of Helsinki, and the data came from an RCT(ChiCTR2100044492).

An experienced therapist performed all clinical measures. FMA-UE with a score of 0–66, was used to assess the severity of motor dysfunction. The patient information, such as name, gender, course of the disease, brain damage, et al. were all recorded in detail.

### EEG acquisition

Participants were asked to sit in a chair in front of a computer screen. An EEG cap was used to record EEG signals. Ten channels of Ag/AgCl electrodes were distributed according to the 10–20 system. The reference channel and the ground channel were placed on the right mastoid process and the forehead, respectively. The impedance of electrodes was kept < 5 kΩ. EEG signals were amplified with the CommercialAmp (iRecorder W16, Niantong Intelligence Ltd., China) and recorded at a sampling rate of 500 Hz.

### Feature extraction and classification

In this study, a Common spatial pattern [49] was used for feature extraction, and two pairs of feature patterns were selected for classification [50]. Then the method of linear discriminative analysis was employed for discriminating different tasks (MA vs. Rest). The pattern classifications were conducted online with 10 channels of EEG signals. EEG features were extracted from the time segment of [3 5] s and frequency band of [8 30] Hz.

### Motor task evaluation

**Fig. 5:**
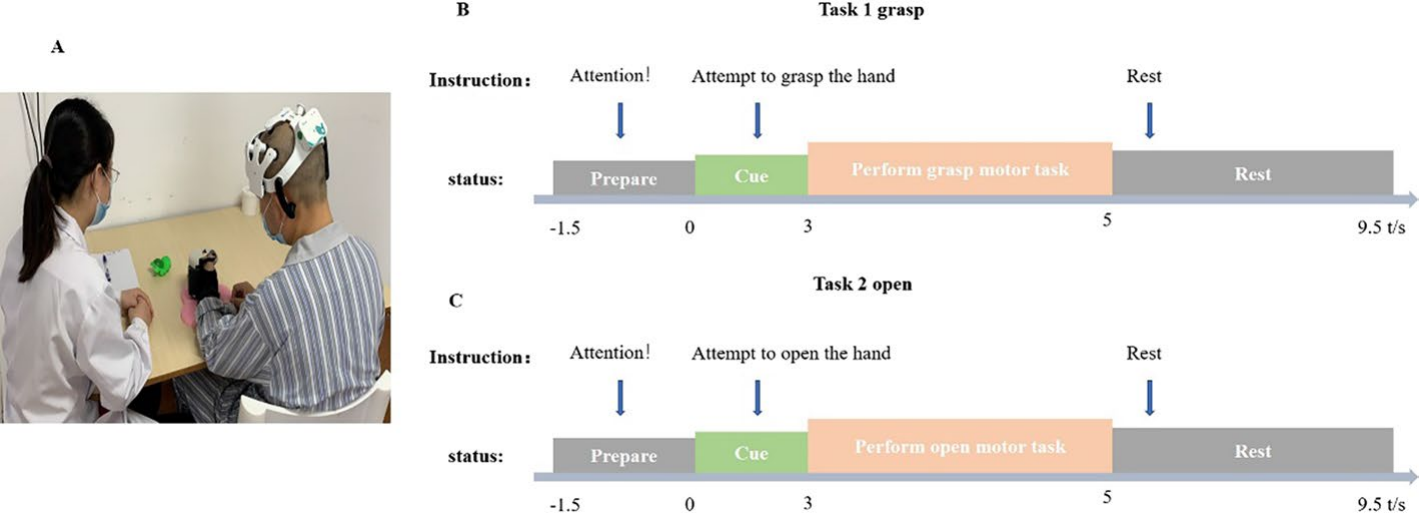
Study setup and experimental protocol. **A** The patient was seated in a chair in front of a desk. **B** Timeline of a single trial during the grasping task. **C** Timeline of a single trial during the open task

Patients were instructed to undergo two kinds of MA evaluation, hand grasping, and opening. During grasp evaluation, the voice instructions are’attempt to grasp, rest’, and ‘relax, rest’ each was ten times but appears in random order. After twenty trials for calibration, there was thirty times evaluation for ‘attempt to grasp, rest’; the open evaluation was the same process, the only difference is the instruction: ‘attempt to open, rest’, ‘relax, rest’. The patients were wearing a robot, so the rhythms in the sensorimotor area of the brain were detected to control the opening and grasping of a robotic hand in the thirty times evaluation for each task, therefore, the feedback can be considered as BCIs accuracy (Fig. 5).

### EEG processing

**Fig. 6:**
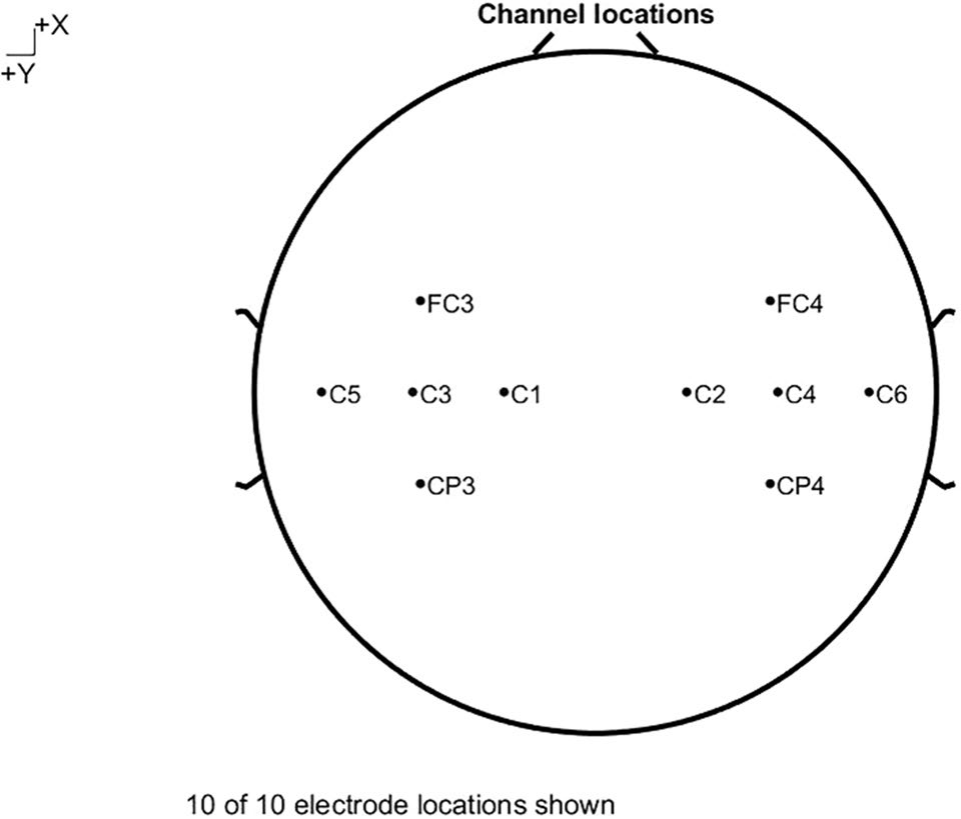
10 of 10 electrode locations shown, the left hemisphere was covered with FC3, CP3, C1, C3, and C5 while the right hemisphere was covered with FC4, CP4, C2, C4, and C6

The EEGLAB v2021.1 and MATLAB R2021a were used in the EEG analysis. EEG data from 10 channels were used in processing. The left hemisphere was covered with FC3, CP3, C1, C3, and C5 (5 channels) while the right hemisphere was covered with FC4, CP4, C2, C4, and C6 (5 channels) (Fig. 6). The preprocessed EEG data consisted of high-pass filtering at 8 Hz and low-pass filtering at 40 Hz. Band-pass filtering was 48–52 Hz. Manual checking was performed in the EEG data of all 10 channels and all trials.

The power spectrum of all 10 channels was computed at the frequency of alpha (8–13 Hz) to identify ERD on grasp and open tasks. Time–frequency distributions of EEG trials were estimated using a windowed Fourier transform (WFT) with a fixed 400 ms Hanning window. WFT yielded, for each trial, a complex time–frequency estimate F(*t*, *f*) at each time–frequency point (*t*, *f*), extending from − 1500 to 8500 ms (in steps of 2 ms) in the time domain, and from 8 to 40 Hz (in steps of 1 Hz) in the frequency domain. Power spectrum (*P*), *P*(*t*, *f*) =|*F*(*t*, *f*)|2, was obtained. The percentage of relative power change was calculated to obtain the ERD concerning a resting-state baseline ([− 1.3, − 0.2] s). The interest time was set both at [3, 5] s after the cue[0, 2]s of the event. During the [3, 5] s, the patient was performing the MA tasks. The power spectrum of interest in the period after the event is given by A whereas that of the preceding baseline period is given by *R*. ERD or ERS was calculated according to the equation:

$${\text{ERD or ERS}} = \left( {A \, {-} \, R} \right)/R.$$

Under this definition, ERD was usually expressed as a negative value, while ERS was expressed as a positive value. The time–frequency maps were drawn with the above-mentioned calculation, representing the signal magnitude as a joint function of time and frequency at each time–frequency point. The topographies were drawn with an interesting time of 3–5 s, concerning a resting state baseline ([− 1.3, − 0.2] s). We calculate the ERD from the affected hemisphere (C3/C4) of the brain and obtain the average power spectrum for every 30 trials.

### Statistical analysis

The statistical analysis was performed with SPSS version 26.0 (SPSS Inc.) and figures were drawn with GraphPad Prism 8 (GraphPad Software, Inc.). Time–frequency graph was drawn by EEGLAB v2021.1. Data conforming to a normal distribution were expressed as mean ± standard deviation, and median (P25–P75) was used for data that did not conform to normal distribution. The chi-square test was used for binary variables; the *t*-test was used for data conforming to normal distribution; otherwise, a non-parametric test was used for basic feature descriptions. Gender (male/female), age, diagnosis (infarction/hemorrhage), MMSE, and course of disease were set as covariables, calculating the partial correlation between the ERD in grasp task and open task with FMA-UE, respectively. In addition, the partial correlation between FMA-UE scores and BCI accuracy was also calculated. According to the Brunnstrom recovery stages, and the hand functional characteristics, the patients were divided into two groups, with group 1 defined with relatively poorer hand function (Brunnstrom recovery stages between I to II) (*n* = 19) which they cannot move or slightly flex fingers, and group 2 with a relatively better hand function (Brunnstrom recovery stages between III to VI) (*n* = 23) which they can grasp or open their hand. Independent-sample *t*-tests were used in the same task for each group, and the results were drawn with a histogram. Statistical significance was set at *P* < *0.05*, two-tailed.

## Data Availability

The dataset analyzed during the current study is available from the corresponding authors upon reasonable request.

## References

[1] Wu S, Wu B, Liu M, Chen Z, Wang W, Anderson CS, Sandercock P, Wang Y, Huang Y, Cui L, Pu C, Jia J, Zhang T, Liu X, Zhang S, Xie P, Fan D, Ji X, Wong KL, Wang L (2019). Stroke in China: advances and challenges in epidemiology, prevention, and management. Lancet Neurol.

[2] Kwah LK, Harvey LA, Diong J, Herbert RD (2013). Models containing age and NIHSS predict recovery of ambulation and upper limb function six months after stroke: an observational study. J Physiother.

[3] Morris JH, van Wijck F, Joice S, Donaghy M (2012). Predicting health related quality of life 6 months after stroke: the role of anxiety and upper limb dysfunction. Disabil Rehabil.

[4] Baniqued PDE, Stanyer EC, Awais M, Alazmani A, Jackson AE, Mon-Williams MA, Mushtaq F, Holt RJ (2021). Brain–computer interface robotics for hand rehabilitation after stroke: a systematic review. J NeuroEng Rehabil.

[5] Cervera MA, Soekadar SR, Ushiba J, Millan J, Liu M, Birbaumer N, Garipelli G (2018). Brain–computer interfaces for post-stroke motor rehabilitation: a meta-analysis. Ann Clin Transl Neurol.

[6] Biasiucci A, Leeb R, Iturrate I, Perdikis S, Al-Khodairy A, Corbet T, Schnider A, Schmidlin T, Zhang H, Bassolino M, Viceic D, Vuadens P, Guggisberg AG, Millán JDR (2018). Brain-actuated functional electrical stimulation elicits lasting arm motor recovery after stroke. Nature Commun.

[7] Wolpaw JR (2013). Brain–computer interfaces. Handb Clin Neurol.

[8] Robinson N, Thomas KP, Vinod AP (2018). Neurophysiological predictors and spectro-spatial discriminative features for enhancing SMR-BCI. J Neural Eng.

[9] Li L, Wang Y, Zeng Y, Hou S, Huang G, Zhang L, Yan N, Ren L, Zhang Z (2021). Multimodal neuroimaging predictors of learning performance of sensorimotor rhythm up-regulation neurofeedback. Front Neurosci.

[10] Pinter D, Kober SE, Fruhwirth V, Berger L, Damulina A, Khalil M, Neuper C, Wood G, Enzinger C (2021). MRI correlates of cognitive improvement after home-based EEG neurofeedback training in patients with multiple sclerosis: a pilot study. J Neurol.

[11] Yuan H, He B (2014). Brain–computer interfaces using sensorimotor rhythms: current state and future perspectives. IEEE Trans Biomed Eng.

[12] Frenkel-Toledo S, Bentin S, Perry A, Liebermann DG, Soroker N (2014). Mirror-neuron system recruitment by action observation: effects of focal brain damage on mu suppression. Neuroimage.

[13] Pfurtscheller G, Lopes DSF (1999). Event-related EEG/MEG synchronization and desynchronization: basic principles. Clin Neurophysiol.

[14] Hasegawa K, Kasuga S, Takasaki K, Mizuno K, Liu M, Ushiba J (2017). Ipsilateral EEG mu rhythm reflects the excitability of uncrossed pathways projecting to shoulder muscles. J NeuroEng Rehabil.

[15] Remsik AB, Williams L, Gjini K, Dodd K, Thoma J, Jacobson T, Walczak M, McMillan M, Rajan S, Young BM, Nigogosyan Z, Advani H, Mohanty R, Tellapragada N, Allen J, Mazrooyisebdani M, Walton LM, van Kan PLE, Kang TJ, Sattin JA, Nair VA, Edwards DF, Williams JC, Prabhakaran V (2019). Ipsilesional Mu Rhythm desynchronization and changes in motor behavior following post stroke BCI intervention for motor rehabilitation. Front Neurosci.

[16] Norman SL, McFarland DJ, Miner A, Cramer SC, Wolbrecht ET, Wolpaw JR, Reinkensmeyer DJ (2018). Controlling pre-movement sensorimotor rhythm can improve finger extension after stroke. J Neural Eng.

[17] Kilteni K, Andersson BJ, Houborg C, Ehrsson HH (2018). Motor imagery involves predicting the sensory consequences of the imagined movement. Nat Commun.

[18] Antelis JM, Montesano L, Ramos-Murguialday A, Birbaumer N, Minguez J (2017). Decoding upper limb movement attempt from EEG measurements of the contralesional motor cortex in chronic stroke patients. IEEE Trans Biomed Eng.

[19] Meng H, Pi Y, Liu K, Cao N, Wang Y, Wu Y, Zhang J (2018). Differences between motor execution and motor imagery of grasping movements in the motor cortical excitatory circuit. PeerJ.

[20] Chen C, Zhang J, Belkacem AN, Zhang S, Xu R, Hao B, Gao Q, Shin D, Wang C, Ming D. G-causality brain connectivity differences of finger movements between motor execution and motor imagery. J Healthc Eng. 2019; 1–12. 10.1155/2019/5068283 PMC6791225 31662834

[21] Bai Z, Fong KNK, Zhang JJ, Chan J, Ting KH (2020). Immediate and long-term effects of BCI-based rehabilitation of the upper extremity after stroke: a systematic review and meta-analysis. J NeuroEng Rehabil.

[22] Naghdi S, Ansari NN, Mansouri K, Hasson S (2010). A neurophysiological and clinical study of Brunnstrom recovery stages in the upper limb following stroke. Brain Inj.

[23] Barios JA, Ezquerro S, Bertomeu-Motos A, Nann M, Badesa FJ, Fernandez E, Soekadar SR, Garcia-Aracil N (2019). Synchronization of slow cortical rhythms during motor imagery-based brain-machine interface control. Int J Neural Syst.

[24] Pichiorri F, Morone G, Petti M, Toppi J, Pisotta I, Molinari M, Paolucci S, Inghilleri M, Astolfi L, Cincotti F, Mattia D (2015). Brain–computer interface boosts motor imagery practice during stroke recovery. Ann Neurol.

[25] Ramos-Murguialday A, Broetz D, Rea M, Läer L, Yilmaz Ö, Brasil FL, Liberati G, Curado MR, Garcia-Cossio E, Vyziotis A, Cho W, Agostini M, Soares E, Soekadar S, Caria A, Cohen LG, Birbaumer N (2013). Brain–machine interface in chronic stroke rehabilitation: a controlled study. Ann Neurol.

[26] Li M, Liu Y, Wu Y, Liu S, Jia J, Zhang L (2014). Neurophysiological substrates of stroke patients with motor imagery-based brain–computer interface training. Int J Neurosci.

[27] Zhang JJ, SánchezVidaña DI, Chan JN, Hui ESK, Lau KK, Wang X, Lau BWM, Fong KNK (2023). Biomarkers for prognostic functional recovery poststroke: a narrative review. Front Cell Dev Biol.

[28] Tang CW, Hsiao FJ, Lee PL, Tsai YA, Hsu YF, Chen WT, Lin YY, Stagg CJ, Lee IH (2020). Beta-oscillations reflect recovery of the paretic upper limb in subacute stroke. Neurorehabil Neural Repair.

[29] Rossiter HE, Boudrias MH, Ward NS (2014). Do movement-related beta oscillations change after stroke?. J Neurophysiol.

[30] Hu L, Peng W, Valentini E, Zhang Z, Hu Y (2013). Functional features of nociceptive-induced suppression of alpha band electroencephalographic oscillations. J Pain.

[31] Chen S, Shu X, Jia J, Wang H, Ding L, He Z, Brauer S, Zhu X (2021). Relation between sensorimotor rhythm during motor attempt/imagery and upper-limb motor impairment in stroke. Clin EEG Neurosci.

[32] Santisteban L, Térémetz M, Bleton J, Baron J, Maier MA, Lindberg PG (2016). Upper limb outcome measures used in stroke rehabilitation studies: a systematic literature review. PLoS ONE.

[33] Kaiser V, Daly I, Pichiorri F, Mattia D, Müller-Putz GR, Neuper C (2012). Relationship between electrical brain responses to motor imagery and motor impairment in stroke. Stroke.

[34] Plow EB, Cunningham DA, Varnerin N, Machado A (2015). Rethinking stimulation of the brain in stroke rehabilitation. Neuroscientist.

[35] Salinas E, Thier P (2000). Gain modulation: a major computational principle of the central nervous system. Neuron.

[36] Chance FS, Abbott LF, Reyes AD (2002). Gain modulation from background synaptic input. Neuron.

[37] Aono K, Miyashita S, Fujiwara Y, Kodama M, Hanayama K, Masakado Y, Ushiba J (2013). Relationship between event-related desynchronization and cortical excitability in healthy subjects and stroke patients. Tokai J Exp Clin Med.

[38] Schwarz A, Ofner P, Pereira J, Sburlea AI, Müller-Putz GR (2017). Decoding natural reach-and-grasp actions from human EEG. J Neural Eng.

[39] Xu B, Zhang D, Wang Y, Deng L, Wang X, Wu C, Song A (2021). Decoding different reach-and-grasp movements using noninvasive electroencephalogram. Front Neurosci.

[40] Wadden KP, Asis KD, Mang CS, Neva JL, Peters S, Lakhani B, Boyd LA (2017). Predicting motor sequence learning in individuals with chronic stroke. Neurorehabil Neural Repair.

[41] Shadmehr R, Krakauer JW (2008). A computational neuroanatomy for motor control. Exp Brain Res.

[42] Haar S, Donchin O (2020). A revised computational neuroanatomy for motor control. J Cogn Neurosci.

[43] Raghavan P, Krakauer JW, Gordon AM (2006). Impaired anticipatory control of fingertip forces in patients with a pure motor or sensorimotor lacunar syndrome. Brain.

[44] Ono T, Shindo K, Kawashima K, Ota N, Ito M, Ota T, Mukaino M, Fujiwara T, Kimura A, Liu M, Ushiba J (2014). Brain-computer interface with somatosensory feedback improves functional recovery from severe hemiplegia due to chronic stroke. Front Neuroeng.

[45] Soekadar SR, Birbaumer N, Slutzky MW, Cohen LG (2015). Brain–machine interfaces in neurorehabilitation of stroke. Neurobiol Dis.

[46] Remsik A, Young B, Vermilyea R, Kiekhoefer L, Abrams J, Evander ES, Schultz P, Nair V, Edwards D, Williams J, Prabhakaran V (2016). A review of the progression and future implications of brain-computer interface therapies for restoration of distal upper extremity motor function after stroke. Expert Rev Med Devices.

[47] Chen S, Cao L, Shu X, Wang H, Ding L, Wang S, Jia J (2020). Longitudinal electroencephalography analysis in subacute stroke patients during intervention of brain–computer interface with exoskeleton feedback. Front Neurosci.

[48] Ander R-M (2012). Proprioceptive feedback and brain computer interface (BCI) based neuroprostheses. PLoS ONE.

[49] Ramoser H, Muller-Gerking J, Pfurtscheller G (2000). Optimal spatial filtering of single trial EEG during imagined hand movement. IEEE Trans Rehabil Eng.

[50] Shu X, Chen S, Yao L, Sheng X, Zhang D, Jiang N, Jia J, Zhu X (2018). Fast recognition of BCI-inefficient users using physiological features from EEG signals: a screening study of stroke patients. Front Neurosci.

