# Correction: Correlation between the ERD in grasp/open tasks of BCIs and hand function of stroke patients: a cross‑sectional study

**DOI:** 10.1186/s12938-023-01127-6

**Published:** 2023-06-16

**Authors:** Jianghong Fu, ZeWu Jiang, Xiaokang Shu, Shugeng Chen, Jie Jia

**Affiliations:** 1 Department of Rehabilitation Medicine, Huashan Hospital, Fudan University, 12 Mid-Wulumuqi Road, Jing’an District, Shanghai, 200040 China; 2 School of Mechanical Engineering, Shanghai Jiao Tong University, Shanghai, China; 3 National Clinical Research Center for Aging and Medicine, Huashan Hospital, Fudan University, Shanghai, China; 4 National Center for Neurological Disorders, Shanghai, China

**Correction: BioMedical Engineering OnLine (2023) 22:36** **https://doi.org/10.1186/s12938-023-01091-1**

Following publication of the original article [1], in Table 1 of this article, the data in the headed “*P* values” were inserted wrongly. So, the correct table will be shown as below:

**Table 1:** The characteristic of the patients from the two groups

Item	Group 1 (*n* = 19)	Group 2 (*n* = 23)	*t*/*χ*/*Z*	*P* values
Age (MD ± SD)	59.21 ± 11.23	54.04 ± 12.06	1.425	0.162
Male/(%)	14 (73.7%)	17 (73.9%)	0.000	> 0.999
Type			0.505	0.477
Infarction	13 (68.4%)	19 (82.6%)		
Hemorrhage	6 (31.6%)	4 (17.4%)		
Course/days	80.00 (42.00, 121.00)	68.00 (26.00, 174.00)	− 0.531	0.596
MMSE	27.00 (24.00, 29.00)	28.00 (26.00, 30.00)	− 1.357	0.175
FMA-UE	10.16 ± 3.82	33.35 ± 13.40	− 7.917	< 0.001

^The data conformed to Gaussian distribution were been demonstrated by MD ± SD, or they were demonstrated by M (P25−P75). *MMSE* mini-mental state examination, *FMA-UE* the upper limb of the Fugl-Meyer assessment^

The original article has been corrected.
